# The *PTPN2* rs1893217 IBD risk allele increases susceptibility to AIEC invasion by a JAK-STAT-CEACAM6 axis

**DOI:** 10.1080/19490976.2025.2526136

**Published:** 2025-07-07

**Authors:** Pritha Chatterjee, Vinicius Canale, Stephanie J. King, Ali Shawki, Hillmin Lei, Alina N. Santos, Michael Haddad, Casey Gries, Dermot P. B. McGovern, James Borneman, Declan F. McCole

**Affiliations:** a Division of Biomedical Sciences, University of California, Riverside, Riverside, CA, USA; b Department of Biology, University of California, Riverside, Riverside, CA, USA; c Department of Gastroenterology, Cedars-Sinai Medical Center, Los Angeles, CA, USA; d Department of Microbiology and Plant Pathology, University of California, Riverside, CA, USA

**Keywords:** AIEC, LF82, JAK-STAT, gut barrier, IBD, tofacitinib

## Abstract

Inflammatory bowel disease (IBD) patients often exhibit expansion of the gut pathobiont, adherent-invasive E. coli (AIEC). Loss of activity of the IBD susceptibility gene, protein tyrosine phosphatase type 2 (PTPN2), causes gut microbiota dysbiosis in IBD patients, while Ptpn2 knock-out (Ptpn2-KO) mice display AIEC expansion. CEACAM6, a host cell surface glycoprotein, is exploited by AIEC to attach to and enter intestinal epithelial cells (IECs). Here, we investigated how IEC-specific PTPN2 restricts AIEC invasion. Intestinal biopsies from IBD patients heterozygous (CT) or homozygous (CC) for the PTPN2 SNP (single nucleotide polymorphism) rs1893217 were stained for CEACAM6. HT-29 IECs were transfected with control shRNA (PTPN2-CTL), or a shRNA targeted toward PTPN2 (PTPN2-KD). The rs1893217 SNP was inserted (PTPN2-KI), or a complete knock-out of PTPN2 (PTPN2-KO) was generated, by CRISPR-Cas9 gene editing of Caco-2BBe IECs. Adherence and invasion assays were performed with either the human IBD AIEC isolate, LF82, or a novel fluorescent-tagged mouse adherent-invasive E. coli (mAIEC^red^). IL-6 and the pan-JAK inhibitor tofacitinib were administered to interrogate JAK-STAT signaling. Protein expression was determined by western blotting and densitometry. CEACAM6 expression was elevated in IBD patients carrying the PTPN2 rs1893217 SNP (CT, CC) compared to wildtype (TT) IBD patients. HT-29 and Caco-2BBe cell lines deficient in PTPN2 expressed significantly higher levels of CEACAM6. Further, PTPN2-KI and PTPN2-KO cell lines displayed greater invasion by LF82 and mAIEC^red^. CEACAM6 was further elevated by IL-6 in PTPN2–deficient IECs vs. untreated controls. STAT1 and 3 silencing partially reduced CEACAM6 protein expression. Tofacitinib significantly reduced the elevated CEACAM6 protein expression and the higher AIEC adherence and invasion in PTPN2-KI and PTPN2-KO IECs. Our findings highlight a crucial role for PTPN2 in restricting pathobiont entry into host cells and suggest a role for JAK-inhibitors in mitigating AIEC colonization in IBD-susceptible hosts.

## GRAPHICAL ABSTRACT

**Figure:**
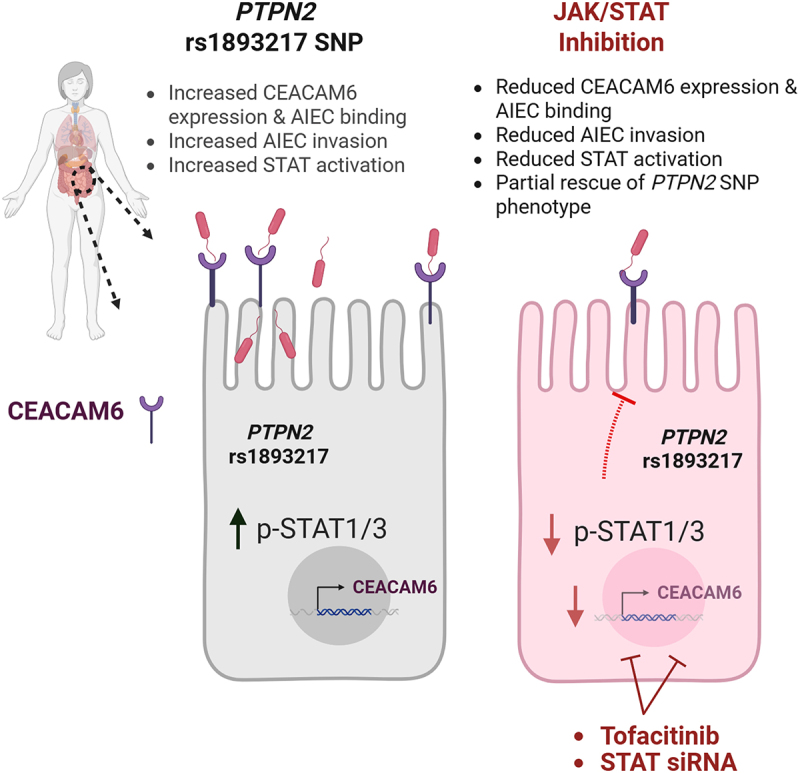
Created in BioRender. McCole, D. (2025) https://biorender.com/undefined.

## Introduction

Inflammatory bowel disease (IBD) encompassing Crohn’s disease (CD), and ulcerative colitis (UC) is a chronic, relapsing condition characterized by inflammation of the gastrointestinal tract.^1–3^ IBD is a multi-factorial condition that can be caused by a faulty immune-system, environmental factors, irregularities in the host microbiome and genetic predispositions.^4,5^ One or more of these factors act in tandem to give rise to this autoinflammatory condition that features an abnormal response of the immune system to the normal intestinal flora, leading to abdominal pain, diarrhea, ulcers, and lesions.^6,7^

Several genome-wide association (GWAS) studies have identified over 240 genes that are associated with IBD.^8^ The single nucleotide polymorphism (SNP) in *rs1893217* present in the non-coding region of the gene protein tyrosine phosphatase type 2 (*PTPN2*), has been associated with several chronic inflammatory conditions like type 1 diabetes (T1D), rheumatoid arthritis (RA) and both subtypes of IBD.^9,10^ The rs1893217 SNP causes loss of functional activity of PTPN2 protein, also referred to as T-cell protein tyrosine phosphatase (TCPTP). The *PTPN2* SNP rs1893217 is carried by 19–20% of IBD patients and 16% of the general population, suggesting increased association in patient cohorts.^11^ *PTPN2* is a negative regulator of several members of the Janus Activated Kinase – signal transducers and activators of transcription (JAK-STAT) pathway, while *PTPN2* expression is increased by pro-inflammatory cytokines IFN-γ and IL-6 in keeping with a negative feedback loop paradigm.^12,13^ Several JAK inhibitors have been approved for clinical use in rheumatoid arthritis and IBD.^14–16^ Tofacitinib (Xeljanz, CP-690,550) is a pan-JAK inhibitor that has been approved for use in patients with moderate-to-severe UC.^17–19^

Along with genetic susceptibilities, altered microbial composition – dysbiosis – has been observed in many IBD patients.^20^ Dysbiosis in IBD is associated with reduced microbial diversity accompanied by a decrease in beneficial commensals like Firmicutes, and a concurrent increase in Proteobacteria and Bacteroidetes.^21^ *PTPN2* SNPs in IBD patients have been identified as modifiers of microbial population dynamics, as well as microbial dysbiosis and increased disease severity.^22,23^ A subset of patients also showed increased abundance of a particular B2 phylogroup of *Escherichia coli* (*E. coli*) – adherent-invasive *E. coli* (AIEC).^24^ The AIEC LF82, initially isolated from the ileal mucosa of a CD patient, has an almost unique ability to attach to and invade intestinal epithelial cells (IECs) and survive within macrophages.^25^ Multiple groups have identified several different AIEC strains associated with both CD and UC patients, highlighting its potential role in disease pathogenesis.^26,27^ Although the exact mechanism by which AIEC contributes to IBD is still unknown, an increasing number of studies have identified its role in the maintenance and/or induction of intestinal inflammation in genetically susceptible hosts.^28–31^

The IECs lining the intestinal mucosa act as a partition between the luminal bacteria and lamina propria immune cells.^32,33^ IECs also maintain a tightly regulated and selectively permeable epithelial barrier. The intestinal epithelium is also responsible for absorption of nutrients and secretion of antimicrobial peptides by enterocytes or specialized ileal crypt IECs (Paneth cells) that prevent pathogenic bacteria from penetrating the mucosal surface and provoke immune cell-mediated inflammatory responses. However, pathobionts like AIEC can utilize certain host proteins in a susceptible host to colonize the intestinal mucosa. A well-described interaction between AIEC and the human host is mediated by carcinoembryonic antigen-related cell-adhesion molecule 6 (CEACAM6) and AIEC-LF82 fimbriae protein FimH.^34,35^ CEACAM6 is a highly mannosylated glycosyl phosphatidylinositol (GPI)-anchored protein that is expressed on the apical surface of IECs and directly interacts with AIEC to facilitate entry into epithelial cells.^36^

Previously, our lab has demonstrated that *Ptpn2-*KO mice exhibit microbial dysbiosis with a profound decrease in Firmicute levels and increased abundance of Proteobacteria compared to wildtype littermate controls.^37^ This increase in Proteobacteria most prominently featured a dramatic expansion of a novel mouse adherent-invasive *E. coli* (*m*AIEC) that genetically overlapped with the human AIEC LF82.^37^ Having identified that loss of PTPN2 caused an expansion of AIEC, the aim of this study was to determine the role of intestinal epithelial *PTPN2* in limiting AIEC invasion of host cells.

## Materials and methods

### *Cells culture and generation of* PTPN2-*KI and KO cell lines*

Caco-2BBe1 cells and HT-29cl.9A IECs were cultured in Dulbecco’s modified Eagle’s Medium (DMEM; Corning, Tewksbury, MA) and McCoy’s 5A medium (Corning, Tewksbury, MA), respectively. The medium was supplemented with 10% heat-inactivated fetal bovine serum (FBS; Gibco, Waltham, MA), 1% L-glutamine (Invitrogen, Carlsbad, CA), and 1% penicillin/streptomycin (Corning, Tewksbury, MA) at 37°C incubator maintained at 5% CO_2_/air mix. Cells were cultured in 6-well plates for protein assay, or in 12-well plates for immunofluorescence. For STAT1 and STAT3 silencing, the cells were transfected with previously validated, STAT1 and STAT3-specific, or non-targeting control siRNA constructs (Dharmacon, Lafayette, CO) using DharmaFECT transfection agents. In experiments with STAT1 and STAT3 siRNA challenged with IL-6, the culture media was replaced with a serum-free medium 8 hr prior to the addition of IL-6 (50 ng/ml; Peprotech, Cranbury, NJ). Twenty-four hours post-IL-6 addition, cells were washed with PBS and collected with RIPA as previously described. In experiments with tofacitinib, the cells were treated with tofacitinib (50 μM, MedChemExpress, Monmouth Junction, NJ). Control cells were treated with an equal amount of vehicle (dimethyl sulfoxide, DMSO, 0.5%, Sigma-Aldrich).

For *PTPN2* knockdown in HT-29 cells, lentiviral particles containing scrambled shRNA (*PTPN2-*CTL) or *PTPN2*-specific shRNA (*PTPN2*-KD) were generated as previously described.^38^ Insertion of SNP *rs1893217* (*PTPN2*-KI) and complete knockout of *PTPN2* (*PTPN2*-KO) in Caco-2BBe cell lines was performed using CRISPR-Cas9 gene editing by Synthego (Menlo Park, CA), as initially reported.^39^ The guide RNAs for PTPN2-KI and KO cell lines are as follows: 5’ AUUCCUAGGGACAAAGGUAG 3’ and 5’ UGUCAUGCUGAACCGCAUUG 3’. For KI and KO cell lines, the guide RNA was designed targeting the gene of interest after meeting the strict off-target requirements of at least 2 mismatches. For KI cell lines, the donor strand 5’TCATCTGCAGGGGCCCAGATACACTCTTCTTCCTCTACCTCTGTCCCTAGGAATGGTGACAAGTGAGAGCACAGGAAACATAT 3’ included homology arms of sufficient lengths to increase the efficiency of knock-ins. The g-RNAs were complexed with sp-Cas9 as ribonucleoproteins (RNPs). The RNPs and donor sequence were electroporated into cells. For the KO cells, only g-RNAs and sp-Cas9 were electroporated into cells. The KI and KO edited regions were PCR amplified, and Sanger sequenced. The edited pool was used to seed single cells for clonal expansion. The seeded cells were monitored every 2–3 days to ensure the population was truly clonal and only progeny of a single cell. The resulting clones were verified using Sanger sequencing.

### Bacterial infection studies

AIEC LF82 was grown in LB broth and *m*AIEC^red^ was grown in chloramphenicol supplemented LB media, overnight at 37°C with 250–300 rpm shaking. The overnight culture was refreshed by adding fresh LB broth and bacterial OD was measured. Caco-2 cells were grown in 24-well plates until 80% confluent and bacteria was added at multiplicity of infection (MOI) 10/cell. For bacterial adherence assays, LF82 or an mCherry-tagged mouse AIEC (*m*AIEC^red^) – original *m*AIEC strain was isolated from *Ptpn2*-KO mice^37^- was allowed to adhere for 3 hrs and then plated on LB agar plates. For bacterial invasion assays, bacteria were allowed to infect cells for 4 hours and then washed with PBS. Fresh DMEM was added with 100 μl/ml of gentamycin and incubated for an additional hour. The cells were then lysed and plated on LB agar plates or LB-chloramphenicol plates. For CEACAM6 blocking experiments, anti-CEACAM6 antibody (Abcam #78029) was added to cell culture medium at a concentration of 10 μl/ml an hour prior to infection with AIEC LF82 or *m*AIEC^red.^ For experiments with tofacitinib, tofacitinib was added to the cell culture media at the concentration of 50 μM for 2 hours and then bacterial infection protocol was performed as mentioned above.

### RNA sequencing and bioinformatic analysis

RNA was isolated from HT-29 CTL and *PTPN2*-KD cells using the Qiagen RNAeasy kit (Qiagen, Valencia, CA). Ribosomal RNA (rRNA) was depleted using the Ribo-Gone rRNA removal kit (Shiga, Japan). Libraries were constructed using the SMARTer Stranded RNA-Seq kit (Clontech, Mountainview, CA). rRNA-depleted RNA was fragmented and converted to double stranded cDNA. Adapters were ligated and the ~300 base pair (bp) long fragments were then amplified by PCR and selected by size exclusion. Each library was prepared with a unique indexed primer for multiplexing. To ensure proper sizing, quantitation, and quality prior to sequencing, libraries were analyzed on the Agilent 2100 Bioanalyzer. Multiplexed libraries were subjected to single-end 100 bp sequencing using the Illumina HiSeq2500 platform.

Data analysis was performed with the RNA-seq workflow module of the systemPipeR package available on Bioconductor24. RNA-Seq reads were demultiplexed, quality filtered and trimmed. Quality reports were generated with the seeFastq function. The genome sequence and annotation from Ensembl (GRCh38.85), was used for genome annotation. RNA-Seq reads were mapped with the alignment suite Bowtie2/Tophat2 against the human reference genome (hg38). Raw expression values in the form of gene-level read counts were generated with the summarizeOverlaps function, counting only the reads overlapping exonic regions of genes, and discarding reads mapping to ambiguous regions of exons from overlapping genes. Normalization and statistical analysis of differentially expressed genes (DEGs) was performed using the edgeR package. Host DEGs were defined as those with a fold change ≥2 and a false discovery rate (FDR) corrected p-value ≤0.05. Reads were normalized by reads per kilobase of transcript per million mapped reads (RPKM). Heatmap was generated using R packages “DESeq2” and “gplots”.

### Immunofluorescence

Caco-2BBe IECs were seeded at 200,000 on coverslips in 12-well plates. After 24 hours, media was deprived of serum and antibiotics. For bacterial fluorescence assay, cells were washed with PBS (x3) followed by fixation with 4% paraformaldehyde (PFA) for 20 min at room temperature. Cells were then permeabilized with 0.3% Triton X-100 (Fisher Scientific, Waltham, MA) for 5 min followed by blocking with 5% Bovine Serum Albumin (BSA; Fisher Scientific, Waltham, MA) for 10 min at room temperature. Cells were incubated with Alexa Fluor 488-Phalloidin antibody (1:1000) (Abcam #176553, Cambridge, MA) for 90 min at room temperature followed by nuclei staining with 4,6-diamidino-2-phenylindole (DAPI; Vector Laboratories, Newark, CA). For CEACAM6 staining, cells were fixed with 4% PFA and permeabilized with Triton X-100 for 30 minutes, followed by blocking with 5% BSA for 1 hour. Cells were then incubated with anti-CEACAM6 antibody (1:100; Abcam #78029; Cambridge, MA) at 37°C for 2 hours. Cells were washed with 0.1% tween supplemented PBS (X3) after which they were incubated in Alexa-Fluor 488 (1:100; #711–586–154, Jackson Research, West Grove, PA) for 1 hour. Images were captured using Leica DM5500 microscope attached with a DFC365 FX camera using a 63X oil immersion objective, or with an inverted Zeiss Airyscan. DAPI was visualized using a 405 nm excitation laser and DAPI filter set. mCherry was visualized using a 561 nm excitation laser and mCherry filter set. Images were analyzed using ImageJ software (NIH).

### Protein isolation and western blotting

For protein isolation from cells, the cells were washed with ice-cold PBS and lysed in radioimmunoprecipitation assay (RIPA) buffer containing phosphatase and protease inhibitors (Roche, South San Francisco, CA). All samples were then sonicated for 30 seconds, centrifuged (10 min. at 13,000 G at 4°C), and the supernatant transferred into fresh tubes. Protein concentration was detected using a BCA assay (Thermo Fisher Scientific, Waltham, MA). For Western blots, aliquots with equal amounts of protein were separated by electrophoresis on polyacrylamide gels, and the proteins blotted on nitrocellulose membranes. The membranes were then incubated in blocking buffer (5% milk, 1% BSA in tris-buffered saline with 0.5% Tween) for 1 hr. and incubated overnight at 4°C with appropriate antibody. The next day, the membranes were washed in tris-buffered saline with 0.5% Tween, incubated with HRP-coupled secondary antibodies (Jackson Immunolabs, West Grove, PA), washed again, and immunoreactive proteins detected using ECL substrate (Thermo Fisher Scientific, Waltham, MA) and X-ray films (GE Healthcare, Chicago, IL)

### Immunohistochemistry of patient biopsies

Microscopy slides with paraffin-embedded and formalin-fixed (PEFF) intestinal biopsies from *PTPN2* genotyped patients (rs1893217) were provided by Dr. Dermot McGovern at Cedars Sinai Medical Center, Los Angeles, CA, USA. Twelve samples from colon (6 WT, 5 Het, 1 KO), and 12 samples from ileal segment (6 WT, 6 KO) were used in this analysis (Suplementary Table S1).^38^ Slides were deparaffinized, rehydrated and processed for immunohistochemistry as described before. Briefly, heat-induced antigen retrieval was performed for 20 minutes at ~ 96 C with sodium-citrate (pH 6) buffer. Primary antibody (Abcam #ab78029, Cambridge, UK) diluted in PBS with 5% NDS at 1:200 dilution was incubated overnight at 4°C. Detection was done using the biotin-streptavidin detection system and signal developed by DAB reaction according to manufacturer’s protocol (Cell Signaling Technology #8059, Beverly, MA,). Then, sections were counterstained with hematoxylin and slides were mounted with Permount® and visualized on a Leica microscope model DM5500B with DFC450C camera (Leica – Nussloch, Germany).

### Statistics

Data are represented as the mean of a series of ‘n’ biological repetitions ± standard deviation (SD). Data followed a Gaussian distribution, and variation was similar between groups for conditions analyzed together. Differences between groups were analyzed with one-way or two-way ANOVA. Tukey post-hoc test was used to determine displayed *P-values. P*-values below 0.05 were considered significant. No data points were excluded from the statistical analysis. Statistical analysis was performed using GraphPad Prism version 9 (GraphPad, San Diego, CA).

## Results

### *The* PTPN2 *rs1893217 SNP, and epithelial* PTPN2 *knockdown, promotes CEACAM6 expression*

**Figure 1.**
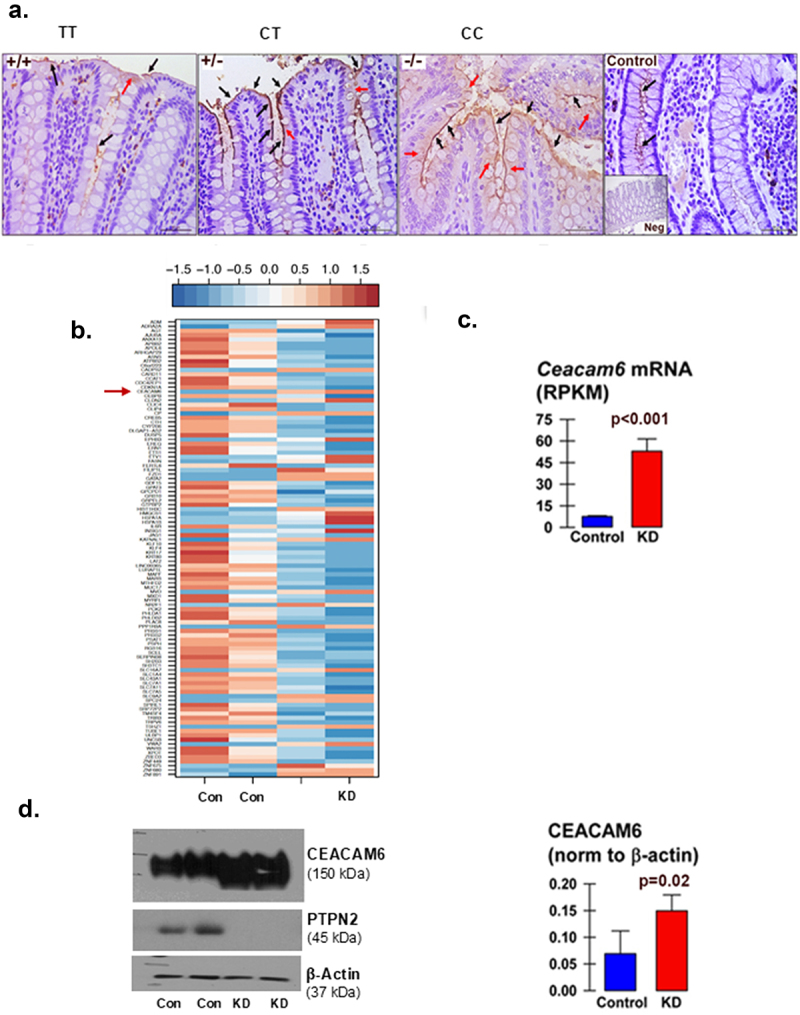
The *PTPN2* rs1893217 SNP and *PTPN2* knockdown promote CEACAM6 protein expression in IBD patients and an intestinal epithelial cell line.

We previously demonstrated that constitutive *Ptpn2-*KO mice displayed microbial dysbiosis and expansion of adherent-invasive *E. coli* (AIEC).^37^ Given that CEACAM6 in IECs is the recognized receptor for AIEC, we wanted to determine the expression of CEACAM6 in the intestinal biopsies of IBD patients genotyped for *PTPN2* loss-of-function SNP *rs1893217*. IBD patients with “TT” wildtype allele were considered controls while, patients with heterozygous “CT” and homozygous “CC”, where “C” is the minor allele carrying the IBD associated SNP, displayed elevated CEACAM6 IEC apical membrane expression in biopsied colonic tissue (Figure 1(a); Supplementary Table S1). Notably, we did not observe any differences in ileal CEACAM6 expression between wildtype carriers and homozygous *PTPN2* SNP carriers (Supplementary Figure S1a). Next, we conducted an RNA sequencing analysis comparing control (*PTPN2*-CTL) and *PTPN2*-KD HT-29cl.9A IEC cell lines (Figure 1(b)). The AIEC-binding receptor, *CEACAM6*, was among the most significantly increased genes in *PTPN2*-KD cells compared to control cells (Figure 1(b,c); Supplementary Table S2). We further confirmed that CEACAM6 protein levels were also significantly elevated in *PTPN2*-KD HT-29 cells compared to control cells (Figure 1(d)). Together, these data suggest that loss of functional epithelial PTPN2 causes intestinal epithelial over-expression of the AIEC receptor protein, CEACAM6, both *in vitro* and in IBD patients.

(a) Colonic biopsies from IBD patients homozygous for the wildtype allele (TT), heterozygous (CT) or homozygous (CC) where ”C” allele possess the *PTPN2* SNP rs1893217 that is associated with IBD susceptibility. Control refers to non-IBD control subjects homozygous for the WT *PTPN2* allele. Biopsies from IBD patients carrying the *PTPN2* variant allele showed higher expression of CEACAM6 (brown). (b) RNA-sequencing analysis from *PTPN2*-CTL and *PTPN2*-KD HT-29 cells with the red arrow indicating higher *Ceacam6* mRNA in the KD condition compared to the control condition. (c) Quantification of *Ceacam6* mRNA. (d) Western blots from control and *PTPN2*-KD HT-29 cells confirm higher CEACAM6 protein expression in PTPN2-KD cells. Actual p-values displayed; Student’s t-test.

### Loss of epithelial PTPN2 increases susceptibility to AIEC invasion

Next, we assessed the functional consequences of increased CEACAM6 in *PTPN2*-deficient cells. To do so, we first generated Caco-2BBe1 cell lines carrying the IBD patient associated SNP *rs1893217* (*PTPN2*-KI) cells and a complete knock-out of the *PTPN2* gene (*PTPN2*-KO) by CRISPR-Cas9 gene editing. As observed in HT-29 cells, we confirmed that expression of CEACAM6 was significantly higher in *PTPN2* deficient (PTPN2-KI and *PTPN2*-KO) IECs compared to control Caco-2BBe cells (*PTPN2*-WT) (Figure 2(a,b)). Interestingly, the expression of CEACAM6 in *PTPN2*-KI cell lines was also higher than in *PTPN2*-KO cells (Figure 2(a,b)). Further, we also stained for CEACAM6 expression in these cells to determine its cellular localization. We confirmed that the overexpression of CEACAM6 observed in *PTPN2-*KI and KO cell lines was primarily found on the cell surface, albeit some was also located intracellularly in all three genotypes (Figure 2(c)). Next, we determined if the increase in CEACAM6 in the KI and KO cells increased susceptibility to AIEC infection. We observed that both *PTPN2-*KI and KO cells displayed increased adherence and invasion by the human AIEC, LF82, compared to the *PTPN2*-WT cells (Figure 2(d,e)). Next, we visually confirmed higher invasion of *PTPN2*-KI and KO cells by using a constitutive mCherry fluorescent-tagged *m*AIEC^red^ (Figure 2(f)). Here we observed some intestinal epithelial cells in all three genotypes were highly invaded by *m*AIEC^red^ as compared to other cells. This phenomenon was more apparent in *PTPN2*-KI and KO cells compared to the control cells. We therefore quantified cells that demonstrated less than 50 bacteria per cell as having “low-level” invasion, while cells that displayed more than 50 bacteria/cell were designated as having a “high level” of invasion. *PTPN2*-KI and *PTPN2*-KO cells displayed a significant increase in susceptibility to both parameters of AIEC invasion compared to control cell lines (Figure 2(g)). We also confirmed increased AIEC LF82 adherence and invasion in *PTPN2*-KD HT-29 IECs compared to controls (Supplementary Figure S2a, b). The *PTPN2*-KD Caco-2BBe’s also demonstrated higher *m*AIEC^red^ invasion compared to *PTPN2*-WT cells (Supplementary Figure S2c). We also identified that *m*AIEC^red^ can interact with the human CEACAM6 (Supplementary Figure S2d). Next, we confirmed a functional role for CEACAM6 in mediating AIEC invasion, by pre-treating cells with a CEACAM6 blocking antibody. The anti-CEACAM6 antibody significantly reduced *m*AIEC^red^ invasion of *PTPN2*-KI and KO cells compared to untreated controls (Figure 2(h)). We also visually confirmed that anti-CEACAM6 reduced *m*AIEC^red^ invasion of epithelial cells (Figure 2(i)). Taken together, these results demonstrate loss of *PTPN2* or expression of a *PTPN2* loss-of-function variant promotes AIEC invasion of host cells by increasing CEACAM6 protein expression.

**Figure 2.**
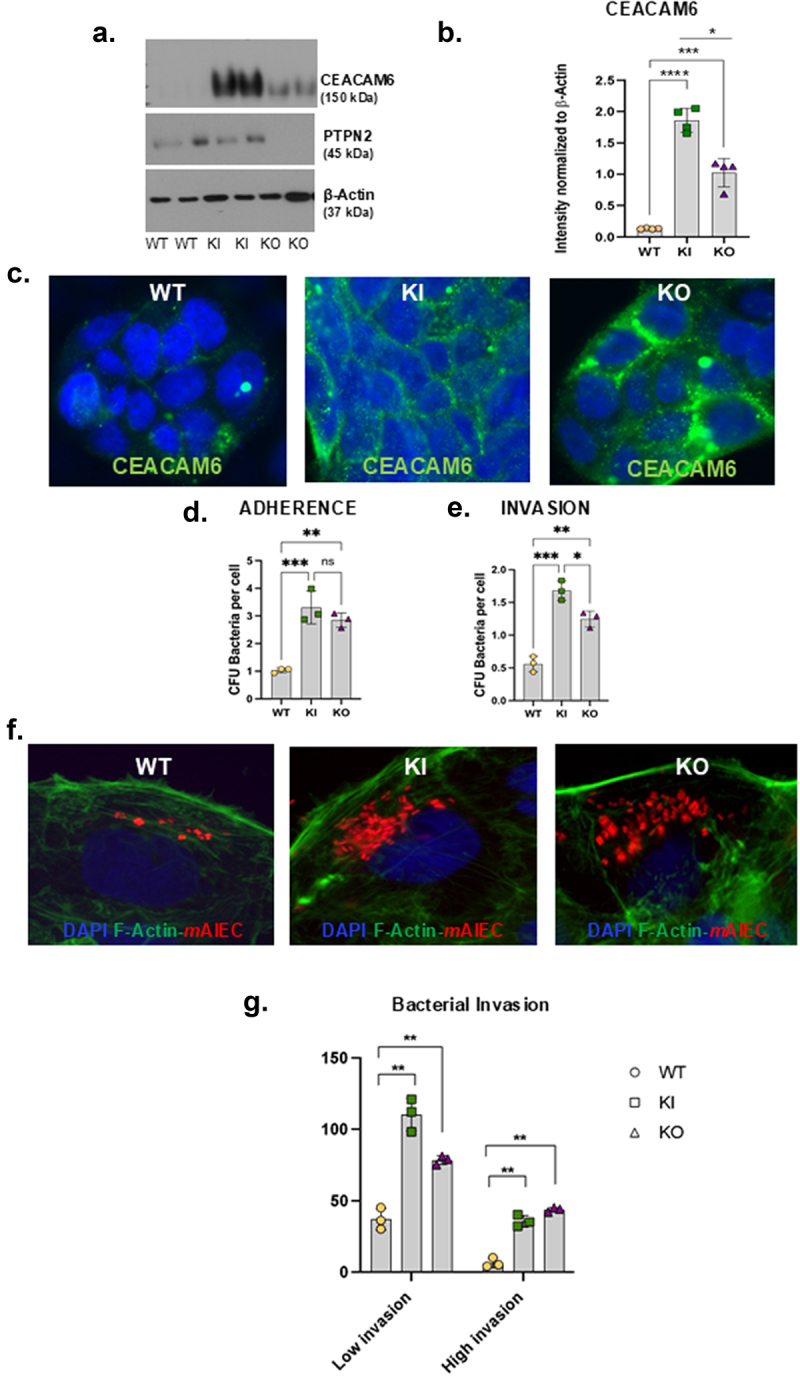
A *PTPN2* loss-of-function variant increases IEC susceptibility to AIEC adherence and invasion.

Caco-2BBe cells were edited by CRISPR-Cas9 to carry the *PTPN2* SNP *rs1893217 (PTPN2*-KI) or a complete knockdown of *PTPN2* gene was generated *(PTPN2*-KO). (a) Representative western blot showing elevated CEACAM6 expression in *PTPN2*-KI and *PTPN2*-KO cells compared to *PTPN2*-WT cells. (b) Densitometric quantification of CEACAM6 protein expression in *PTPN2*-KI and *PTPN2*-KO cells compared to WT cells. (c) *PTPN2*-WT, KI and KO cells were immunostained for CEACAM6. (d) AIEC LF82 adherence; and (e) invasion of *PTPN2*-KI and KO cells compared to controls. (f) Immunofluorescence showing *m*AIEC^red^ invasion of *PTPN2*-KI and KO cells compared to control (WT) cells. (g) We quantified cells that demonstrated less than 50 bacteria per cell as having “low-level” invasion, while cells that displayed more than 50 bacteria/cell were designated as having a “high level” of invasion. *PTPN2*-KI and KO cells exhibited both high and low *m*AIEC^red^ invasion of individual epithelial cells compared to *PTPN2*-WT cells. (h) Anti-CEACAM6 antibody was used to block CEACAM6 protein and its effect on AIEC invasion was determined. (i) Visualization of the effect of anti-CEACAM6 on *m*AIEC^Red^ invasion was determined by immunofluorescence staining. *P* < 0.05 = *; *P* < 0.01 = **; *P* < 0.005 =***; ANOVA with the Tukey post-hoc test.

**Figure 2.**
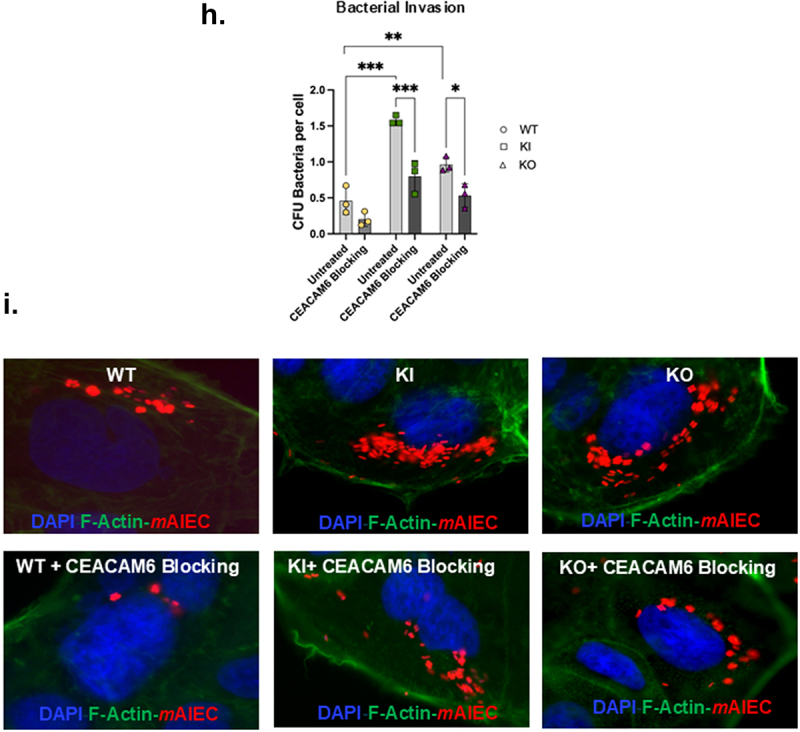
(Continued).

### IL-6 promotes CEACAM6 expression in PTPN2-deficient IECs

Previous studies have shown that CEACAM6 expression can be elevated by pro-inflammatory cytokines such as IL-6 and IFNγ. There also seems to be an interaction between CEACAM6 protein and STAT3, which may indicate a possible STAT3 binding site in the CEACAM6 promoter region (Supplementary Figure S5a). Since PTPN2 is a negative regulator of the JAK-STAT signaling pathway and suppresses IL-6 induced JAK-STAT activation, we assessed whether IL-6 promotes CEACAM6 expression in epithelial cell lines. Twenty-four hours post-IL-6 treatment, both *PTPN2*-KI and *PTPN2*-KO cells displayed higher levels of phosphorylated STAT3 compared to WT cell lines (Figure 3(a)). We also observed that CEACAM6 expression was elevated in *PTPN2*-WT cells after IL-6 treatment and this effect was further exacerbated in *PTPN2*-KI and *PTPN2*-KO cells (Figure 3(a,b)). To determine if the elevated CEACAM6 levels were a consequence of higher STAT3 activation, we performed STAT3 silencing using siRNA constructs. Interestingly, we did not observe a reduction in CEACAM6 levels after STAT3 siRNA transfection, although p-STAT3 levels were dramatically lowered in *PTPN2*-deficient cell lines (Supplementary Figure S5b). Next, we silenced both STAT1 and STAT3 in these cell lines. We observed a decrease in CEACAM6 expression between untreated and IL-6 treated *PTPN2*-KO IECs after STAT1 + STAT3 silencing (Figure 3(c)). We also observed a decrease in CEACAM6 expression of WT cells treated with IL-6 (control siRNA vs. STAT1 + STAT3 silencing), but no changes were observed in *PTPN2*-WT cells treated with control (scrambled) or STAT1 and STAT3 siRNA. Interestingly, in PTPN2-KI cells, we saw no significant impact on CEACAM6 expression after STAT1+STAT3 siRNA treatment (Figure 3(c)). We also confirmed that IFN-γ-STAT1/STAT3 induction elevated the expression of CEACAM6 (Supplementary Figure S4a, b). Together, these data suggest that cytokine-induced CEACAM6 expression is regulated, at least in part, by STAT1 and STAT3 activation, and this mediates the elevated CEACAM6 expression observed in IECs with no PTPN2 activity.

**Figure 3.**
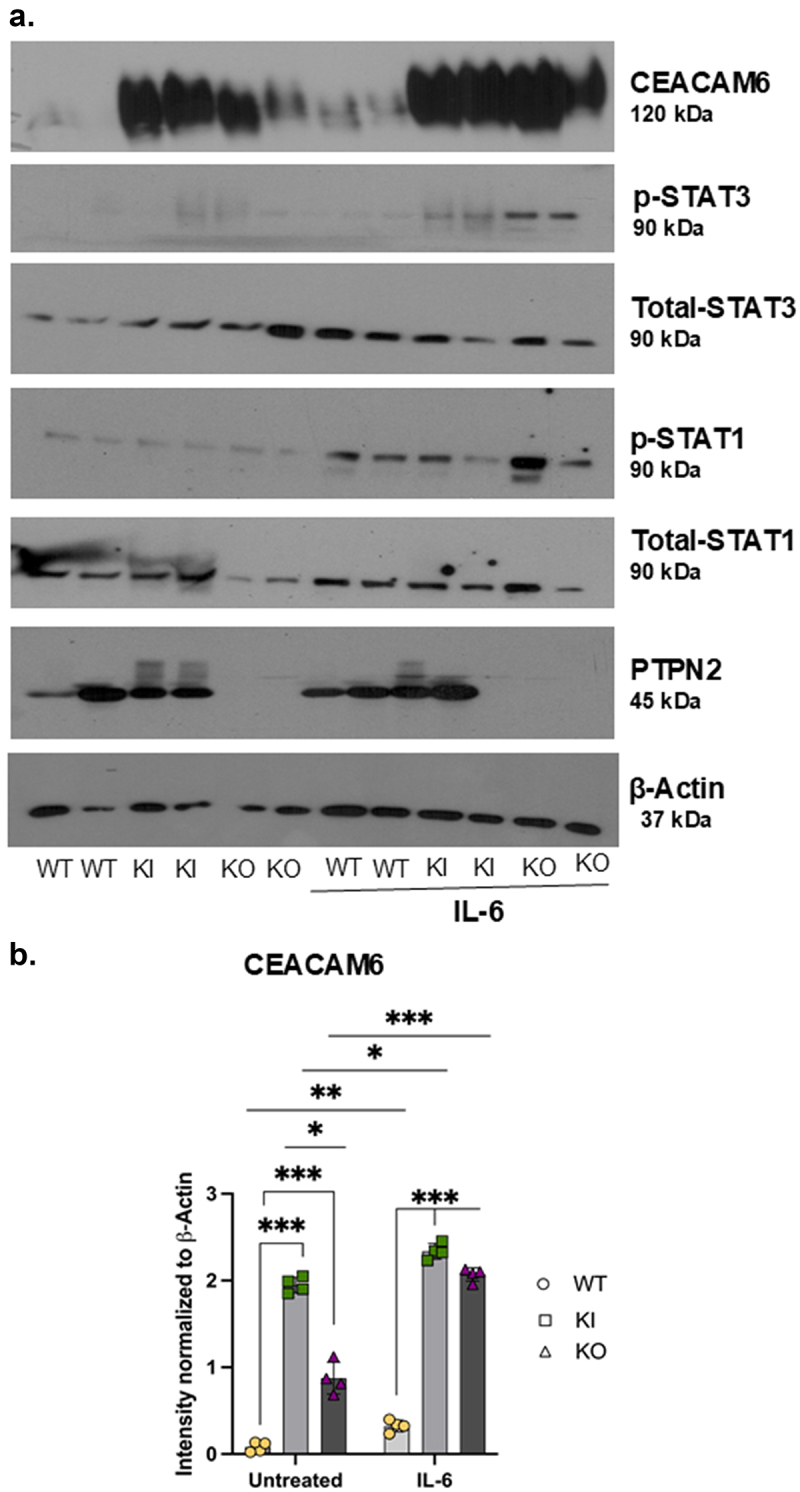
IL-6 promotes CEACAM6 expression in *PTPN2*-deficient Caco-2 BBe cells.

*PTPN2-*WT, KI and KO mice were treated with IL-6 at 50 ng/ml for 24 hours. (a) Representative western blot images depicting β-actin, PTPN2, CEACAM6, total and phosphorylated STAT1 and STAT3. CEACAM6 protein expression. Due to an error in the order of sample loading, the red line demarcates the cropped and flipped portion of the actual blot. (b) Densitometry analysis of CEACAM6 protein expression 24-hours after administration of IL-6. (c) Representative western blot images showing WT, KI and KO cells treated with non-targeting control (siControl) and STAT specific (siSTAT1) and STAT3 specific (siSTAT3) siRNA. After 72 hours, IL-6 was administered and CEACAM6 protein expression was determined. *P* < 0.05 = *; *P* < 0.01 = **; *P* < 0.005 =***. Groups were compared by 2-way ANOVA with Tukey multiple comparisons test.

**Figure 3.**
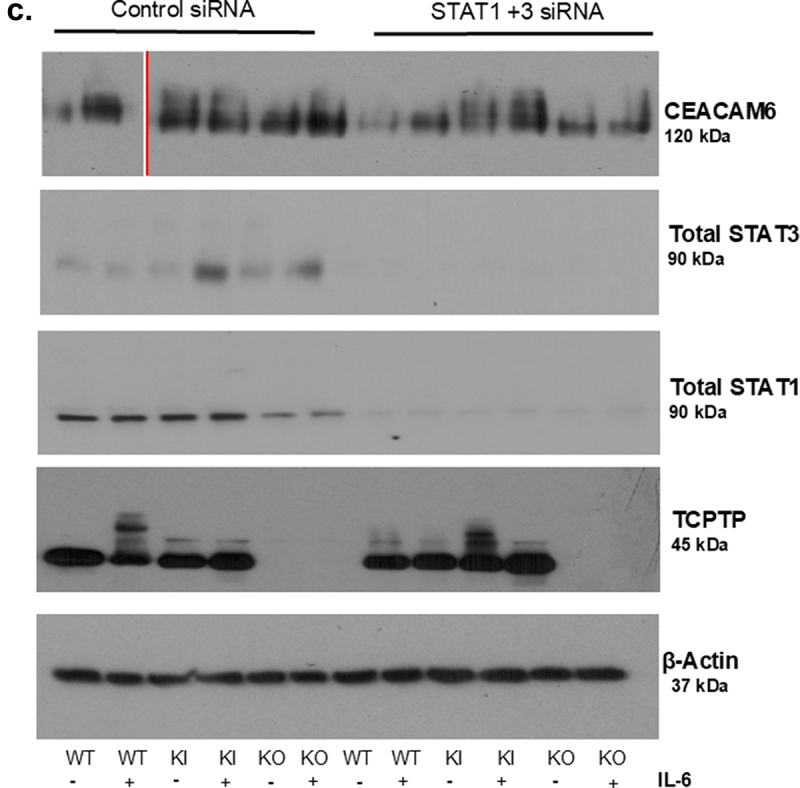
(Continued).

### Tofacitinib rescues CEACAM6 protein overexpression and limits AIEC invasion in PTPN2-deficient IECs

Given that the inhibition of STATs 1 and 3 reduced the over-expression of CEACAM6 in *PTPN2*-KO cell line, we next determined if the FDA approved JAK inhibitor for UC, tofacitinib, could also prevent CEACAM6 over-expression and prevent susceptibility to AIEC infection. All three IEC genotypes were pre-treated with tofacitinib for 2 hours and challenged with IL-6 for another 2 hours. We observed that IL-6 increased CEACAM6 expression in all 3 genotypes (Figure 4(a,b)). Treatment of the KI and KO cell lines with tofacitinib greatly reduced the phospho-STAT1 and STAT3 levels both in basal and IL-6 treatment conditions (Figure 4(a)). Additionally, pre-treatment of *PTPN2*-KO cell lines with tofacitinib reduced CEACAM6 expression in basal conditions and in response to IL-6 stimulated expression (Figure 4(a,b)). The elevated CEACAM6 in *PTPN2*-KI cell lines displayed a moderate, but statistically significant, reduction in CEACAM6 protein expression with tofacitinib treatment in both control and IL-6 challenge conditions (Figure 4(a,b)). Tofacitinib reversed IL-6 induction of CEACAM6 expression in PTPN*2*-WT cells compared to its respective DMSO control condition (Figure 4(a,b)). Interestingly, PTPN2-WT cells treated with tofacitinib displayed an increase in CEACAM6 protein expression (Figure 4(a,b)). We also visually confirmed that tofacitinib reduced CEACAM6 expression in *PTPN2*-KI and KO cell lines (Figure 4(c)). Next, we determined if treatment with tofacitinib also reduced susceptibility to AIEC infection in *PTPN2-*deficient cell lines. We observed that *PTPN2-*WT cells showed no change in AIEC adherence or invasion in the presence of tofacitinib. However, both *PTPN2*-KI and KO cells show reduced AIEC adherence and invasion in the presence of tofacitinib (Figure 4(d,e)). Together, these results suggest that tofacitinib can, at least in part, correct the overexpression of CEACAM6 and increased AIEC invasion found in cells with reduced PTPN2 activity.

**Figure 4.**
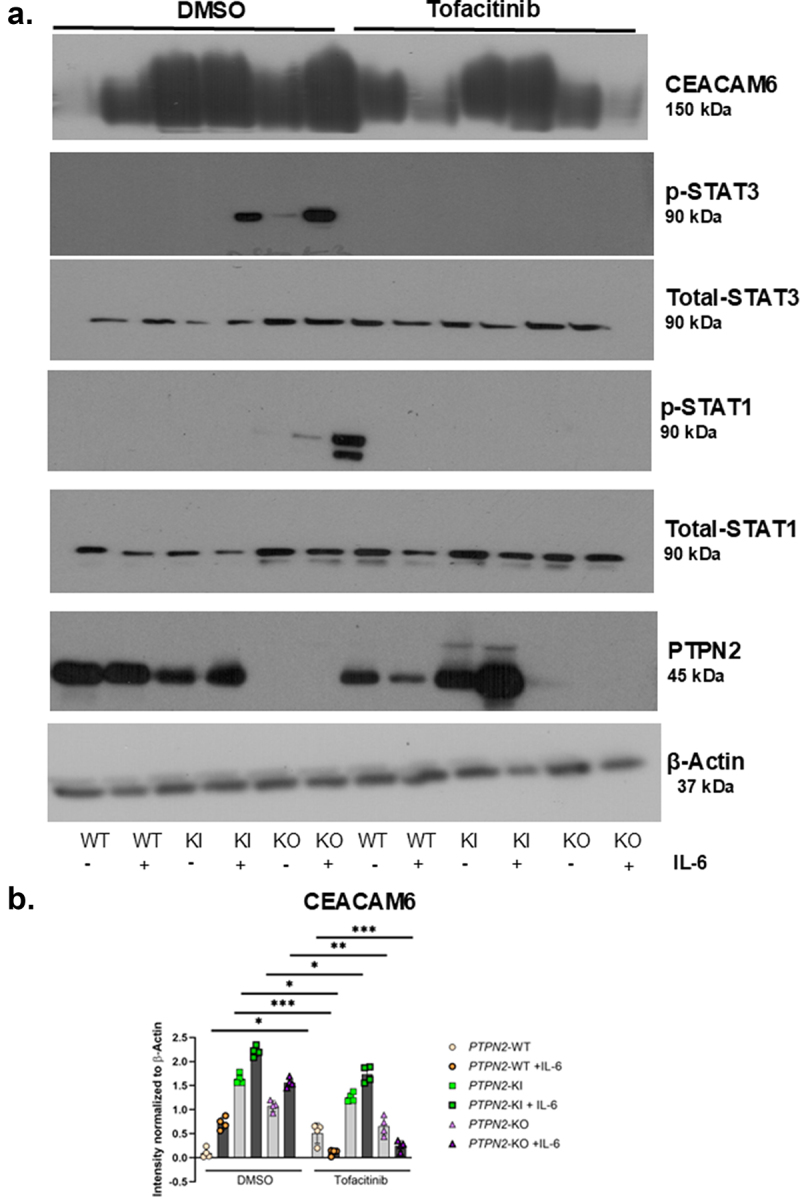
Tofacitinib rescues CEACAM6 overexpression and limits AIEC invasion in *PTPN2* deficient IECs.

Cells were pre-treated with DMSO or tofacitinib at 50 μg/ml for 2 hours and challenged with IL-6 at 50 ng/ml for 2 hours. (a). Representative western blot images depicting β-actin, PTPN2, CEACAM6, phosphorylated and total STAT1 and STAT3. (b). Densitometric analysis of CEACAM6 protein following DMSO or tofacitinib pre-treatment in *PTPN2* WT, KI and KO cell lines. (c). CEACAM6 (green) staining in *PTPN2*-WT,KI and KO cells treated with DMSO or tofacitinib. (d). Adherence E. Invasion of AIEC-LF82 in *PTPN2*-KI and KO cell lines after tofacitinib treatment compared to DMSO controls. *P* < 0.05 = *; *P* < 0.01 = **; *P* < 0.005 =***. Groups were compared by 2-way ANOVA with Tukey multiple comparisons test.

**Figure 4.**
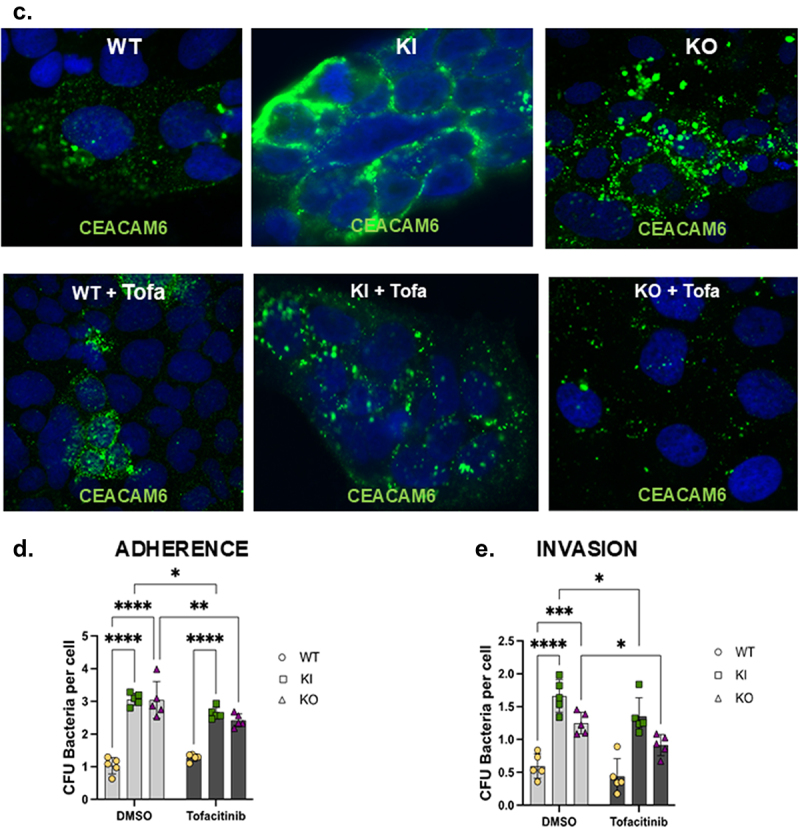
(Continued).

## Discussion

Previously, our lab demonstrated that loss of whole-body PTPN2 in mice causes expansion of an AIEC pathobiont.^37^ In this current study, we describe that the loss of epithelial PTPN2 increases the susceptibility to AIEC invasion by increasing the expression of CEACAM6, a critical receptor for AIEC that mediates entry into IECs. Further, we have proposed a unique role of the UC approved drug, tofacitinib (Xeljanz) in alleviating CEACAM6 expression in *PTPN2*-deficient cell lines and limiting susceptibility to AIEC invasion. Taken together, these data highlight a multifactorial role for *PTPN2* in maintaining microbial homeostasis and restricting bacterial entry into host cells.

In this study, we showed that CEACAM6 was significantly upregulated in *PTPN2* deficient colonic epithelial cell lines, where PTPN2 expression was stably knocked down, or CRISPR/Cas9 edited to either express the clinical *PTPN2* rs1893217 loss-of-function variant or delete PTPN2. Further, IBD patients who are heterozygous (CT) or homozygous (CC) for the *PTPN2* susceptibility variant *rs1893217*, displayed increased colonic CEACAM6 expression compared to *PTPN2* wildtype (TT) controls. Interestingly, we did not observe any differences in CEACAM6 expression in the ileum between wildtype and homozygous *PTPN2* SNP carriers. Recent studies have shown that AIEC has been associated in both CD (55.5%) and UC (35.7%) patients.^40^ Further, CEACAM6 is increased in the colonic biopsies of both CD and UC patients.^36^ While the lack of difference in ileal CEACAM6 expression between healthy and *PTPN2* genotyped patients will be part of our follow-up study, our existing data do provide novel insights into how *PTPN2* variants affect patient pathophysiology and the receptor for AIEC in both small and large intestine.

A complicating feature of AIEC is that it is difficult to distinguish them from other *E. coli* pathotypes as a molecular marker unique to AIEC is yet to be identified. The field has therefore relied on phenotypic adherence/invasion assays and macrophage survival assays to functionally designate an *E. coli* as an AIEC.^41,42^ Given these constraints, no large-scale study has yet identified expansion of AIEC in IBD patients carrying *PTPN2* SNPs. Moreover, it is not possible to study the *PTPN2* regulation of CEACAM6 using an *in vivo* murine model since there is no murine homologue of CEACAM6. Considering these limitations, we generated Caco-2BBe cell lines carrying the disease-relevant *PTPN2* variant *rs1893217* or in which PTPN2 was deleted. We validated that the increased expression of CEACAM6 is of functional consequence by demonstrating that *PTPN2*-variant carrying (*PTPN2*-KI), or PTPN2 deletion (*PTPN2*-KO), cell lines have increased AIEC adherence and invasion burden. Moreover, suppression of increased phosphorylated STAT1 and STAT3 partially reduced the over-expression of CEACAM6 in *PTPN2*- WT and KO cell lines challenged with IL-6, thus functionally confirming the mechanism by which reduced *PTPN2* activity, and cytokine treatment, increases CEACAM6 expression. Curiously, we observed increased CEACAM6 expression in WT Caco-2BBe cells without exogenous stimuli after tofacitinib treatment. It is likely that expression of JAK-STAT proteins is necessary for the homeostatic functioning of intestinal epithelial cells. Several labs have reported basal activation of JAKs and STATs without any exogenous stimuli in different cell types. A study in our lab reported that tofacitinib treatment of WT Caco-2BBe epithelial cells in the absence of any exogenous stimuli, completely repressed basal tyrosine phosphorylation of STAT1/3.^43^ Although, a thorough study has not been undertaken regarding the homeostatic JAK-STAT signaling pathway in intestinal epithelial cells, it has been shown that baseline JAK-STAT signaling is necessary for maintaining the characteristic transcriptional and epigenetic homeostatic state in macrophages and T-cells.^44^ It is therefore possible that suppression of basal JAK-STAT signaling by tofacitinib changes the transcriptional pattern of epithelial cells under homeostatic conditions and, by yet unclear mechanisms, this may result in an increase in CEACAM6 expression in these cell lines. Further, a reduction of CEACAM6 expression was not observed in the *PTPN2*-KI cell lines, indicating that other pathways are likely involved in these cell lines that potentiate the overexpression of CEACAM6. Moreover, *PTPN2*-KI cells were less responsive to CEACAM6 induction by the JAK-STAT activating cytokines, IL-6 and IFN-y, than cells in which PTPN2 had been deleted. This suggests that *PTPN2*-KI cells had a lower dynamic range of response to these cytokines, at least with respect to CEACAM6 induction, than *PTPN2*-KO cells. Further translational relevance was supplied by showing that pretreatment with the JAKi, tofacitinib, reduced CEACAM6 expression in *PTPN2*-KO cells, whereas the CEACAM6 overexpression in *PTPN2*-KI cells was reduced by tofacitinib to a smaller extent. These differences in the suppression of CEACAM6 by a JAKi in *PTPN2*-KI vs. *PTPN2*-KO cell lines may be due to differences in the mechanisms of CEACAM6 regulation in the two genotypes. It is possible that CEACAM6 overexpression is controlled by pathways other than JAK-STAT signaling in *PTPN2-*KI cells and thus its expression was not drastically reduced with tofacitinib. We also observed that in addition to being overexpressed, CEACAM6 protein is highly glycosylated in *PTPN2*-KI cells. While we can only speculate as to the mechanisms involved at this point, it is possible that in the *PTPN2*-KI cell line either the involvement of additional transcription factors, or disproportionate epithelial cell glycosylation, may somehow limit the capacity of tofacitinib to restrict CEACAM6 expression. The roles of both of these factors in governing CEACAM6 regulation in *PTPN2*-KI cells will be an area of interest for our future studies.

Using murine models, our lab has demonstrated that mice lacking PTPN*2* in macrophages (*Ptpn2*-LysMCre) are more susceptible to AIEC invasion and exhibit increased bacterial translocation to extra-intestinal organs that can be blocked in vivo with an anti-mouse CEACAM1 antibody.^45^ Further, a CEACAM6 blocking antibody prevented AIEC (LF82) invasion of human *PTPN2*-deficient macrophages, demonstrating that CEACAM6 also mediates AIEC entry of human macrophages as well. In addition, we identified that mice with IEC-specific knockout of *Ptpn2* (*Ptpn2*^∆IEC^) display higher AIEC tissue burden and increased intestinal barrier permeability compared to control groups (submitted in a separate manuscript to *Gut Microbes*). Altogether, our findings have identified an important role for PTPN2 in limiting AIEC pathogenesis.

CEACAM6 can be upregulated by several inflammatory cytokines, while the AIEC itself can upregulate CEACAM6 expression to promote its own tissue colonization.^36^ Despite its high association with disease, very few therapeutic strategies have been investigated to prevent AIEC colonization. Recently, a study demonstrated that the anti-TNF medication, adalimumab, restricted AIEC replication in CD macrophages by inducing Flotillin (FLOT-1) and restricting chitinase 3-like 1 proteins (CHI3L1), an AIEC receptor, in macrophages.^46^ In the current study, we have demonstrated for the first time how an IBD-susceptibility *PTPN2* gene variant regulates a critical cell surface receptor to mediate AIEC colonization. We further demonstrate that the consequences of reduced PTPN2 activity can be mitigated with the FDA approved JAK inhibitor, tofacitinib. These data shed further light on additional cellular and mechanistic targets of JAKi that may contribute to overall therapeutic benefit. Our findings further suggest that tofacitinib, and potentially other JAK1 or JAK3 targeting drugs, may prove particularly effective in treating patients carrying loss-of-function *PTPN2* SNPs not only with respect to promoting mucosal barrier healing but also to restrict pathobiont colonization.

## Supplementary Material

Suppl_Table_1_Patient_Characteristics_FINAL.docx

Supplementary_Table_2_HT_29_RNAseq.docx
