# Supplementary material for: The PTPN2 rs1893217 IBD risk allele increases susceptibility to AIEC invasion by a JAK-STAT-CEACAM6 axis
Source: Gut Microbes. 2025 Jul 7;17(1):2526136. doi: 10.1080/19490976.2025.2526136 (PMC12239772; doi:10.1080/19490976.2025.2526136)
Supplement: Suppl_Table_1_Patient_Characteristics_FINAL.docx [file KGMI_A_2526136_SM8061.docx]

**Supplementary Table 1. Patient characteristics**

| Variant | Diagnosis | Region | Sex | Age Range |
| --- | --- | --- | --- | --- |
| AA/TT | CD | Colon | F | 45-50 |
| AA/TT | CD | Colon | M | 20-25 |
| AA/TT | CD | Colon | F | 40-45 |
| AA/TT | CD | Colon | F | 70-75 |
| AA/TT | CD | Colon | F | 30-35 |
| AA/TT | CD | Colon | F | 20-25 |
| GA/CT | CD | Colon | M | 70-75 |
| GA/CT | CD | Colon | F | 15-20 |
| GA/CT | CD | Colon | F | 45-50 |
| GA/CT | CD | Colon | F | 45-50 |
| GA/CT | CD | Colon | F | 70-75 |
| GG/CC | CD | Colon | M | 30-35 |
| AA/TT | CD | Ileum | F | 45-50 |
| AA/TT | CD | Ileum | M | 20-25 |
| AA/TT | CD | Ileum | F | 40-45 |
| AA/TT | CD | Ileum | F | 70-75 |
| AA/TT | CD | Ileum | F | 30-35 |
| AA/TT | CD | Ileum | F | 20-25 |
| GG/CC | CD | Ileum | F | 45-50 |
| GG/CC | CD | Ileum | M | 25-30 |
| GG/CC | CD | Ileum | F | 35-40 |
| GG/CC | CD | Ileum | M | 25-30 |
| GG/CC | CD | Ileum | F | 35-40 |
| GG/CC | CD | Ileum | M | 40-45 |

**Supplementary Table 1: Colonic tissues from CD patients harboring the loss-of-function IBD-associated *PTPN2* rs1893217 SNP.**

Adapted from: Marchelletta, R. R., M. Krishnan, M. R. Spalinger, T. W. Placone, R. Alvarez, A. Sayoc-Becerra, V. Canale, A. Shawki, Y. S. Park and L. H. Bernts (2021). "T cell protein tyrosine phosphatase protects intestinal barrier function by restricting epithelial tight junction remodeling." The Journal of Clinical Investigation 131(17).
