# Supplementary material for: The PTPN2 rs1893217 IBD risk allele increases susceptibility to AIEC invasion by a JAK-STAT-CEACAM6 axis
Source: Gut Microbes. 2025 Jul 7;17(1):2526136. doi: 10.1080/19490976.2025.2526136 (PMC12239772; doi:10.1080/19490976.2025.2526136)
Supplement: Supplementary_Table_2_HT_29_RNAseq.docx [file KGMI_A_2526136_SM8060.docx]

**Supplementary Table 2. Bulk RNAseq analysis**

| HumanEnsembl | KD.C_logFC | KD.C_logCPM | KD.C_LR | KD.C_PValue | KD.C_FDR | hgnc_symbol |
| --- | --- | --- | --- | --- | --- | --- |
| **ENSG00000157240** | 3.577182445 | 1.0250814 | 20.1812987 | 7.04E-06 | 0.00316118 | **FZD1** |
| **ENSG00000278272** | 3.281669142 | 2.14562595 | 34.7775025 | 3.70E-09 | 6.08E-06 | **HIST1H3C** |
| **ENSG00000047457** | 3.109806193 | 0.84253402 | 12.8871146 | 0.00033085 | 0.04146438 | **CP** |
| **ENSG00000081803** | 3.106136283 | 1.26924739 | 18.5613651 | 1.65E-05 | 0.00566642 | **CADPS2** |
| **ENSG00000086548** | 2.769585684 | 6.52016416 | 130.278424 | 3.56E-30 | 2.64E-26 | **CEACAM6** |
| **ENSG00000179348** | 2.646355837 | 1.57592962 | 19.0946524 | 1.24E-05 | 0.00484805 | **GATA2** |
| **ENSG00000197372** | 2.642893642 | 1.24359106 | 12.501279 | 0.00040667 | 0.04857125 | **ZNF675** |
| **ENSG00000175745** | 2.4554035 | 2.63591491 | 28.5735259 | 9.02E-08 | 8.35E-05 | **NR2F1** |
| **ENSG00000102781** | 2.260022523 | 2.10157147 | 12.8002652 | 0.00034657 | 0.04207135 | **KATNAL1** |
| **ENSG00000158528** | 1.910769764 | 2.35745082 | 17.2629127 | 3.25E-05 | 0.00852593 | **PPP1R9A** |
| **ENSG00000150594** | 1.868884388 | 3.29535041 | 18.4978808 | 1.70E-05 | 0.00572519 | **ADRA2A** |
| **ENSG00000161888** | 1.743059262 | 4.20270252 | 31.8326396 | 1.68E-08 | 2.07E-05 | **SPC24** |
| **ENSG00000006468** | 1.657694822 | 2.64060974 | 14.4248223 | 0.00014587 | 0.02400321 | **ETV1** |
| **ENSG00000118596** | 1.605749916 | 2.73831027 | 15.4151905 | 8.63E-05 | 0.0175066 | **SLC16A7** |
| **ENSG00000173041** | 1.603759525 | 2.48624463 | 13.4729208 | 0.00024203 | 0.03413793 | **ZNF680** |
| **ENSG00000148926** | 1.544233257 | 2.72333467 | 13.3713097 | 0.0002555 | 0.03514798 | **ADM** |
| **ENSG00000214029** | 1.509032068 | 3.45936214 | 14.2691698 | 0.00015844 | 0.02496254 | **ZNF891** |
| **ENSG00000186480** | 1.490847123 | 5.6285992 | 18.9488902 | 1.34E-05 | 0.00497125 | **INSIG1** |
| **ENSG00000115616** | 1.489972383 | 2.77013572 | 14.0558066 | 0.00017747 | 0.02709544 | **SLC9A2** |
| **ENSG00000168386** | 1.44099699 | 4.15213084 | 16.8315837 | 4.08E-05 | 0.0100826 | **FILIP1L** |
| **ENSG00000165816** | 1.313881897 | 3.67916957 | 16.2340956 | 5.60E-05 | 0.01257825 | **VWA2** |
| **ENSG00000204389** | 1.257160505 | 6.68337586 | 17.2474253 | 3.28E-05 | 0.00852593 | **HSPA1A** |
| **ENSG00000179981** | 1.254547996 | 3.77437572 | 15.6872163 | 7.47E-05 | 0.01585139 | **TSHZ1** |
| **ENSG00000182580** | 1.252659003 | 4.94257103 | 20.5185966 | 5.91E-06 | 0.00281751 | **EPHB3** |
| **ENSG00000165376** | 1.237433669 | 7.37962567 | 26.3555008 | 2.84E-07 | 0.00022138 | **CLDN2** |
| **ENSG00000204388** | 1.187086885 | 6.74476335 | 14.9824073 | 0.00010852 | 0.01971906 | **HSPA1B** |
| **ENSG00000167508** | 1.186343684 | 3.9011145 | 14.4856874 | 0.00014123 | 0.02350106 | **MVD** |
| **ENSG00000112972** | 1.112685085 | 7.75890539 | 14.9260376 | 0.00011181 | 0.01971906 | **HMGCS1** |
| **ENSG00000169710** | 1.062102539 | 8.92772179 | 13.606716 | 0.00022538 | 0.03304797 | **FASN** |
| **ENSG00000128283** | -1.002624185 | 6.60052039 | 12.8197887 | 0.00034297 | 0.04207135 | **CDC42EP1** |
| **ENSG00000149150** | -1.011231162 | 6.11804388 | 18.3135224 | 1.87E-05 | 0.00609359 | **SLC43A1** |
| **ENSG00000155090** | -1.01317718 | 6.38578541 | 14.9031324 | 0.00011318 | 0.01971906 | **KLF10** |
| **ENSG00000166986** | -1.018323817 | 6.99886041 | 17.6185448 | 2.70E-05 | 0.00768819 | **MARS** |
| **ENSG00000137962** | -1.029458715 | 8.64417595 | 13.5671875 | 0.00023017 | 0.03342036 | **ARHGAP29** |
| **ENSG00000134954** | -1.029585897 | 5.65466733 | 14.3654032 | 0.00015054 | 0.02423416 | **ETS1** |
| **ENSG00000169903** | -1.031481341 | 7.20638128 | 18.2286661 | 1.96E-05 | 0.00617319 | **TM4SF4** |
| **ENSG00000167767** | -1.057876614 | 6.29071573 | 14.8194586 | 0.00011831 | 0.02037382 | **KRT80** |
| **ENSG00000146733** | -1.064072895 | 6.55671483 | 20.9605434 | 4.69E-06 | 0.00239432 | **PSPH** |
| **ENSG00000125772** | -1.075666155 | 4.8901413 | 14.0232936 | 0.00018056 | 0.02728666 | **GPCPD1** |
| **ENSG00000262001** | -1.113928312 | 6.24503774 | 19.0137961 | 1.30E-05 | 0.00492819 | **DLGAP1-AS2** |
| **ENSG00000139514** | -1.116722616 | 8.74227192 | 16.5269559 | 4.80E-05 | 0.01127519 | **SLC7A1** |
| **ENSG00000184575** | -1.128721744 | 8.02704284 | 13.2729416 | 0.00026926 | 0.03592618 | **XPOT** |
| **ENSG00000103257** | -1.133760876 | 7.28111845 | 14.2083052 | 0.00016365 | 0.02544215 | **SLC7A5** |
| **ENSG00000138678** | -1.140548127 | 4.45922773 | 13.0949497 | 0.00029609 | 0.03846611 | **GPAT3** |
| **ENSG00000101384** | -1.140753753 | 7.32371979 | 13.510251 | 0.00023726 | 0.03378728 | **JAG1** |
| **ENSG00000164284** | -1.144020655 | 6.82566078 | 17.304665 | 3.18E-05 | 0.00852593 | **GRPEL2** |
| **ENSG00000163697** | -1.151563786 | 5.19667736 | 20.4603924 | 6.09E-06 | 0.00281751 | **APBB2** |
| **ENSG00000140105** | -1.170050954 | 6.66468081 | 17.5319208 | 2.83E-05 | 0.00789469 | **WARS** |
| **ENSG00000146592** | -1.185523228 | 5.94439625 | 16.784221 | 4.19E-05 | 0.01016794 | **CREB5** |
| **ENSG00000132846** | -1.187521793 | 5.66705244 | 17.0285429 | 3.68E-05 | 0.00940234 | **ZBED3** |
| **ENSG00000106070** | -1.202527949 | 6.7492277 | 25.2170578 | 5.12E-07 | 0.00032986 | **GRB10** |
| **ENSG00000169504** | -1.222115797 | 7.1459503 | 25.7284861 | 3.93E-07 | 0.000291 | **CLIC4** |
| **ENSG00000125089** | -1.229079726 | 4.71927907 | 14.9531681 | 0.00011021 | 0.01971906 | **SH3TC1** |
| **ENSG00000135744** | -1.249646074 | 3.76195224 | 14.999965 | 0.00010751 | 0.01971906 | **AGT** |
| **ENSG00000221963** | -1.281147644 | 4.69851935 | 22.3628035 | 2.26E-06 | 0.00123802 | **APOL6** |
| **ENSG00000151012** | -1.285865849 | 8.06020868 | 12.7438433 | 0.00035718 | 0.04300715 | **SLC7A11** |
| **ENSG00000178607** | -1.308065877 | 5.72283573 | 12.9730481 | 0.00031601 | 0.0403454 | **ERN1** |
| **ENSG00000173275** | -1.310593714 | 4.09757354 | 18.8569909 | 1.41E-05 | 0.00508936 | **ZNF449** |
| **ENSG00000172216** | -1.313623861 | 6.04056807 | 12.8014034 | 0.00034636 | 0.04207135 | **CEBPB** |
| **ENSG00000136155** | -1.316164372 | 5.95784485 | 28.7509147 | 8.23E-08 | 8.34E-05 | **SCEL** |
| **ENSG00000185022** | -1.323248106 | 4.81513633 | 17.6526691 | 2.65E-05 | 0.00768819 | **MAFF** |
| **ENSG00000166268** | -1.327355791 | 5.50798857 | 26.6246028 | 2.47E-07 | 0.00020329 | **MYRFL** |
| **ENSG00000111252** | -1.339571116 | 5.03050304 | 25.0947322 | 5.46E-07 | 0.00033682 | **SH2B3** |
| **ENSG00000065911** | -1.349810984 | 8.10351755 | 18.7456547 | 1.49E-05 | 0.00526684 | **MTHFD2** |
| **ENSG00000160712** | -1.350001056 | 3.53547065 | 15.0013807 | 0.00010743 | 0.01971906 | **IL6R** |
| **ENSG00000172432** | -1.350366642 | 6.67066853 | 22.7597775 | 1.84E-06 | 0.00104563 | **GTPBP2** |
| **ENSG00000166401** | -1.381530413 | 3.6069062 | 15.3518376 | 8.92E-05 | 0.01785888 | **SERPINB8** |
| **ENSG00000129474** | -1.383649683 | 6.36162026 | 13.3752993 | 0.00025496 | 0.03514798 | **AJUBA** |
| **ENSG00000059728** | -1.38982151 | 5.7361967 | 17.8586567 | 2.38E-05 | 0.00704762 | **MXD1** |
| **ENSG00000136826** | -1.411462352 | 5.08980983 | 28.701631 | 8.44E-08 | 8.34E-05 | **KLF4** |
| **ENSG00000074935** | -1.446070436 | 4.29879907 | 15.1112324 | 0.00010136 | 0.01949504 | **TUBE1** |
| **ENSG00000138166** | -1.447954956 | 4.38100197 | 18.1623579 | 2.03E-05 | 0.00625873 | **DUSP5** |
| **ENSG00000153714** | -1.463650707 | 3.81150123 | 14.6753685 | 0.0001277 | 0.02173908 | **LURAP1L** |
| **ENSG00000181577** | -1.467390184 | 4.93687763 | 19.7357051 | 8.89E-06 | 0.00387344 | **C6orf223** |
| **ENSG00000115902** | -1.499862687 | 5.74327191 | 15.8529452 | 6.85E-05 | 0.01491017 | **SLC1A4** |
| **ENSG00000115295** | -1.546102938 | 3.3465203 | 19.5949283 | 9.57E-06 | 0.004031 | **CLIP4** |
| **ENSG00000139289** | -1.552392043 | 7.18848807 | 21.9253547 | 2.83E-06 | 0.00149932 | **PHLDA1** |
| **ENSG00000124762** | -1.573761019 | 4.83790827 | 28.376972 | 9.98E-08 | 8.70E-05 | **CDKN1A** |
| **ENSG00000100889** | -1.588062608 | 6.4075385 | 15.6822961 | 7.49E-05 | 0.01585139 | **PCK2** |
| **ENSG00000135069** | -1.604177272 | 7.51978657 | 17.9272447 | 2.30E-05 | 0.00693688 | **PSAT1** |
| **ENSG00000124882** | -1.614334742 | 7.62611461 | 19.5503031 | 9.80E-06 | 0.004031 | **EREG** |
| **ENSG00000128422** | -1.619701827 | 3.42080146 | 13.0578338 | 0.00030202 | 0.03889474 | **KRT17** |
| **ENSG00000134278** | -1.623981711 | 6.01412394 | 34.7815362 | 3.69E-09 | 6.08E-06 | **SPIRE1** |
| **ENSG00000070669** | -1.770899854 | 7.36217382 | 16.6659136 | 4.46E-05 | 0.01064762 | **ASNS** |
| **ENSG00000104537** | -1.775012786 | 6.54062763 | 18.2943508 | 1.89E-05 | 0.00609359 | **ANXA13** |
| **ENSG00000165125** | -1.837421691 | 2.31712063 | 16.2314985 | 5.61E-05 | 0.01257825 | **TRPV6** |
| **ENSG00000086730** | -1.852230822 | 2.96980732 | 14.9058018 | 0.00011302 | 0.01971906 | **LAT2** |
| **ENSG00000143333** | -1.8710306 | 3.92843851 | 20.724981 | 5.30E-06 | 0.00261741 | **RGS16** |
| **ENSG00000214814** | -1.912440047 | 3.18487403 | 25.5127945 | 4.39E-07 | 0.00029801 | **FER1L6** |
| **ENSG00000101255** | -1.916032286 | 7.37391583 | 19.4038717 | 1.06E-05 | 0.00423455 | **TRIB3** |
| **ENSG00000130513** | -1.925665321 | 6.84565415 | 25.49868 | 4.43E-07 | 0.00029801 | **GDF15** |
| **ENSG00000247844** | -1.980558559 | 4.19521762 | 29.4121697 | 5.85E-08 | 6.67E-05 | **CCAT1** |
| **ENSG00000145287** | -2.144438953 | 6.82497822 | 79.3231634 | 5.27E-19 | 1.56E-15 | **PLAC8** |
| **ENSG00000111981** | -2.154263452 | 4.21085061 | 16.355407 | 5.25E-05 | 0.01215025 | **ULBP1** |
| **ENSG00000275896** | -2.290721835 | DEG | 15.1851518 | 9.75E-05 | 0.0189932 | **PRSS2** |
| **ENSG00000116761** | -2.443256979 | 5.47937941 | 41.2034759 | 1.37E-10 | 2.90E-07 | **CTH** |
| **ENSG00000143515** | -2.838938843 | 2.06946996 | 23.1606316 | 1.49E-06 | 0.00088278 | **ATP8B2** |
| **ENSG00000197408** | -2.977557424 | 4.60892275 | 82.0707802 | 1.31E-19 | 4.86E-16 | **CYP2B6** |
| **ENSG00000107731** | -3.008938726 | 3.72203858 | 34.2595789 | 4.82E-09 | 7.14E-06 | **UNC5B** |
| **ENSG00000144824** | -3.622852961 | 2.81750868 | 48.5660451 | 3.19E-12 | 7.88E-09 | **PHLDB2** |
| **ENSG00000198286** | -4.058822386 | 0.39248737 | 13.6072088 | 0.00022532 | 0.03304797 | **CARD11** |
| **ENSG00000204983** | -4.371433333 | 3.55000521 | 84.3694932 | 4.10E-20 | 2.03E-16 | **PRSS1** |
| **ENSG00000169876** | -5.057227523 | 4.23347845 | 138.936142 | 4.55E-32 | 6.74E-28 | **MUC17** |
| **ENSG00000224511** | -5.356934837 | 1.08420357 | 32.2359102 | 1.37E-08 | 1.84E-05 | **LINC00365** |
| **ENSG00000188451** | -7.468502055 | -0.4507903 | 15.6320918 | 7.69E-05 | 0.01590499 | **SRP72P2** |

**Supplementary Table 2. Bulk RNAseq analysis of HT-29 control (wild-type) and PTPN2-knockdown intestinal epithelial cells.** Bulk RNA seq analysis of confluent HT-29 intestinal epithelial cells transfected with control or PTPN2-shRNA. Genes with increased expression are shown in teal; genes with decreased expression are shown in purple.
